# Retrospective analysis of older travellers attending a specialist travel health clinic

**DOI:** 10.1186/s40794-019-0094-8

**Published:** 2019-09-18

**Authors:** Milad Darrat, Gerard T. Flaherty

**Affiliations:** 1 School of Medicine, National University of Ireland Galway, Galway, Ireland; 2 School of Medicine, International Medical University, Kuala Lumpur, Malaysia

**Keywords:** Older traveller, Travel medicine, Travel, Special group of travellers, Elderly

## Abstract

**Background:**

Older people represent a significant proportion of overseas travellers. The epidemiology of older international travellers is not well described in the literature. This study aims to identify demographics, travel characteristics and the medical profile of older travellers seeking pre-travel health advice in a specialist travel medicine clinic.

**Methods:**

Records of travellers aged 60 years and older attending the Tropical Medical Bureau clinic in Galway, Ireland between 2014 and 2018 were examined. Descriptive and inferential.

analysis of data was performed.

**Results:**

A total of 337 older travellers sought pre-travel health advice during the study period. The mean age of the cohort was 65.42 (±10) years. Most of the travellers (*n* = 267, 80%) had at least one travelling companion. Nearly half of older travellers (*n* = 155, 46.8%) were travelling with a single companion. Tourism was the main reason for travel for the majority (*n* = 260, 77.6%), followed by visiting friends and relatives (VFR) (*n* = 23, 6.9%) travellers. The mean interval remaining before the planned trip was 4.36 (±2) weeks, and the mean duration of travel was 3.16 (±1) weeks. The most popular single country of destination was India with 33 (9.8%) visitors, and South East Asia was the most popular region with 132 (39.2%) older travellers. The majority of travellers (*n* = 267, 79.2%) had a documented pre-existing medical condition. The most commonly reported medical conditions were hypertension (*n* = 26, 7.7%), dyslipidaemia (*n* = 18, 5.3%), diabetes mellitus (*n* = 12, 3.5%), insect bite sensitivity (*n* = 11, 3.3%), and hypothyroidism (*n* = 9, 2.6%). Antihypertensive agents (*n* = 32, 9.4%) and statins (*n* = 24, 7.1%) were the most frequently used medications. Typhoid (*n* = 112, 33.2%) and hepatitis A (*n* = 84, 24.9%) were the most common vaccinations administered to older travellers at the clinic.

**Conclusions:**

This study provides an insight into the demographics, travel characteristics, and medical profile of elderly travellers seeking advice at a large travel clinic in Ireland. A wide range of travel destinations, diseases and medication use was reported among this group of travellers, which may enable travel medicine physicians to provide more tailored advice and to more appropriately counsel older travellers.

## Background

The United Nations predicts that the global population will reach 10 billion by 2050, nearly 20% of whom will be aged 60 years or older [1]. This increasing number of older people has implications for travel medicine. Older people represent a significant proportion of all travellers abroad. The World Tourism Organization anticipates that the number of international travellers will approach nearly 2 billion by 2030, with an estimated 15–30% of travellers aged 60 years or older [2]. Many older people have both the desire and capacity to take long overseas trips, especially if they feel well enough to do so [3]. The better quality of life, improved care of chronic illnesses, and financial stability after retirement have led to an increase in travel in this age group [4–8]. However, this can be challenging for health care professionals and services.

The older traveller group tends to have at least one chronic non-communicable disease [9]. Several studies have reported that the most common causes of death abroad were cardiovascular diseases, malignancies and trauma while infectious diseases were responsible for less than 10% of traveller deaths abroad [9–14]. This information implies that underlying non-communicable diseases which occur commonly in the older age group should be optimised in advance of international travel. There are significant physiological and clinical differences between older and younger travellers [15]. Older travellers are at higher risk of travel-associated morbidity and mortality. This is because they are less able to adapt physiologically during journeys and less able to adjust to climatic extremes. This increases the risk of exacerbation of their chronic medical conditions and reduces older people’s immune response to travel vaccines [15].

Chronic medication use and polypharmacy are common issues for older travellers. Co-morbid medical conditions such as hypertension, dyslipidaemia, diabetes mellitus and chronic obstructive pulmonary disease are often coexisting, and each requires a combination of medications [16]. Several studies have reported that long-term medication use could influence the travel duration and itinerary of younger travellers [16–18]. There was no comparison with the older traveller group in these studies. Nonetheless, it was determined that 10–60% of older travellers in a large cohort study were taking at least one chronic medication [19]. Adherence to polypharmacy during travel can be very difficult as a result of time zone changes and travel disruption. This can also lead to exacerbation of underlying medical conditions [20, 21].

Ageing is strongly associated with immunosenescence [22]. Many older people may have poor protection against certain infectious diseases [22]. It has been established that less than half of the 65 years and over population in the USA and Europe have demonstrable antibodies against diphtheria and tetanus [23]. Few studies have examined the vaccination status and vaccine effectiveness of elderly travellers [24, 25]. It has been reported that 30–50% of international travellers of different age groups will suffer accidental injury or seek medical advice during their trip [26]. The cost of travel health insurance for older travellers is typically more expensive than that for younger travellers because of an increased proportion of claims and more costly medical expenses and evacuation, particularly with pre-existing conditions. This can discourage some elderly travallers from purchasing travel insurance and undermine their travel health status [17, 27].

The epidemiology of older travellers, including their characteristics, travel destination, pre-existing medical conditions, use of chronic medication and their vaccination status has not been well addressed by the available literature. Within the European context, limited research has been published that addresses the demographic and clinical profiles of older individuals travelling abroad. In this study, we analysed demographic attributes, travel itinerary, health profile, pre-existing illness and use of chronic medications among a cohort of older travellers who sought pre-travel advice at a specialist travel medicine clinic.

## Methods

### Patients and data source

Patients of 60 years of age and older were identified as older travellers for the purpose of this study [28]. Pre-travel medical registration cards completed between 2014 and 2018 at the Tropical Medical Bureau (TMB), Galway, Ireland were examined. These records had been completed by patients immediately prior to their consultation. They included data on age, gender, nationality, occupation, number in travelling party, travel destination, purpose of travel, duration of travel, interval before date of departure, departure and return dates, duration of stay, medical history including any pre-existing conditions, chronic medications, allergies, vaccination history, and health insurance. The record also includes a panel, which allows travellers to select from a list of common medical conditions. The travel medicine physician verifies these data and adds information on any prescribed antibiotics, antimalarial agents, vaccines or other medications that were prescribed during the clinic visit.

### Data collection and statistical analysis

Data were extracted from medical registration cards and entered into a Microsoft Excel Office 365 Version 1811 database. All entered data were de-identified and assigned corresponding codes. Countries and regions were categorised according to the United Nations Statistics Division [29]. Interval and duration of travel were recorded and quantified in weeks. Medical data were recorded and entered according to a standardised list of 556 possible disease diagnoses [30]. Some patients were assigned to more than one pre-existing medical condition. Numerical variables were analysed using mean and median while categorical variables were reported as frequencies and percentages. Differences between proportions were determined by using a Chi-square test and non-parametric variables were assessed using Wilcoxon-Mann Whitney test. *P*-values were reported for comparisons between the groups of specific interest, with a *p*-value < 0.05 considered statistically significant. No comparison was made between the older traveller and younger traveller groups in our database.

## Results

Of 7123 medical registration cards available, 337 (5%) records related to travellers aged 60 years and over. The mean age of these travellers was 65.42 (±10) years. More than half of the traveller sample (*n* = 179, 53.5%) were above 65 years of age with the remaining travellers (*n* = 155, 46.4%) between 60 and 64 years. Just over half of the travellers were female (*n* = 180, 53.8%). The majority of travellers were from Ireland (*n* = 289, 87.3%), and the United Kingdom (*n* = 28, 8.4%). The remaining countries contributed about 4.2% (*n* = 14) of the older travellers (Table 1).

**Table 1:** Demographics and travel characteristics of older travellers

Characteristics	Frequency (*N*)	Proportion (%)
Age		
Mean Age (Years)	65.42	
60–64 years	155	46.4
> =65 years	179	53.5
Total	334	
Gender		
Male	154	46.1
Female	180	53.8
Total	334	
Country of origin		
Ireland	289	87.3
UK	28	8.4
USA	6	1.8
Germany	2	0.6
Ghana	2	0.6
Philippines	2	0.6
Poland	1	0.3
UAE	1	0.3
Total	331	
Travel alone/accompanied		
Alone	70	21.1
With one	35	10.5
With two	155	46.8
With three	18	5.4
Between 4 and 10	35	10.6
More than 11	18	5.4
Total	331	
Occupation		
Retired	235	69.9
All Employees	54	16.1
Constructors	25	7.4
Medical Practitioners	12	3.6
Others	10	2.9
Total	336	100
Accommodation		
Hotel	279	83.0
Hostel	8	2.4
Camping	5	1.5
Cruise	5	1.5
Others	19	5.7
Unknown	5	1.5
Multiple	15	4.5
Total	336	100
Purpose of travel		
Business	12	3.6
Holiday	260	77.6
Assignment	13	3.9
Trekking	5	1.5
Visiting friends-relatives	23	6.9
Others	1	0.2
Unknown	4	1.1
Multiple	17	5.0
Total	335	100

Most of the travellers were planning to travel with someone during their index trip. Nearly half of them planned on travelling in a pair (*n* = 155, 46.8%). The rest were going to travel in groups of between 3 and 11 persons. Only 70 (21.1%) older persons planned to travel alone. Most travellers (*n* = 235, 69.9%) in our sample were retired from active employment. Employees (teachers, administrators, accountants, health care professionals and social workers) comprised 19.7% (*n* = 66) of older travellers.

Hotel accommodation was the most popular accommodation choice with 83% (*n* = 279) of the travellers planned to choose this form of accommodation. About 2.4% (*n* = 8) of the travellers were going to stay in hostel accommodation. Camping and cruise ships were going to accommodate 1.5% (*n* = 5) each. About 4.5% (*n* = 15) of travellers expressed their intention to stay in multiple forms of accommodation.

The most commonly cited purpose of travel was a holiday in 77.6% (*n* = 260) of the group, followed by visiting friends or relatives (VFR) with 6.9% (*n* = 23) travellers. Just 3.9% (*n* = 13) of travellers were travelling on assignments. Only 1.5% (*n* = 5) of travellers were planning to trek (Table 1). The mean interval between visiting the clinic and travel was 4.364 (±2) weeks. The majority of the travellers (*n* = 331, 98.2%) sought pre-travel advice 1–7 weeks before their departure date. The mean duration of travel was 3.167 (±1) weeks. There was no significant association between the purpose of travel and the time remaining before departure (*p* = 0.8).

**Fig. 1:**
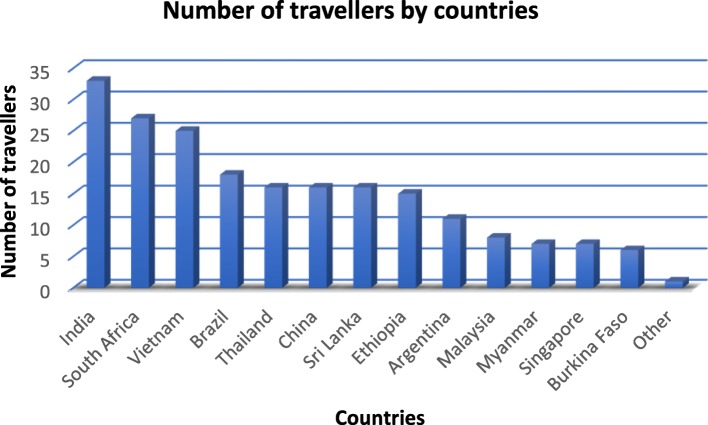
Number of travellers by country

**Fig. 2:**
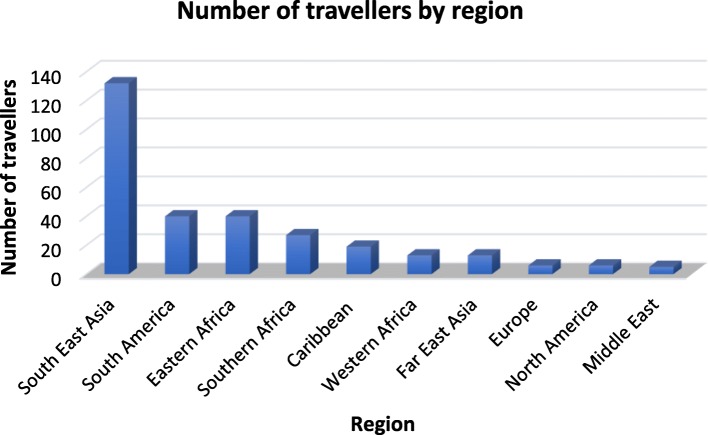
Number of travellers by region

Most of the cohort (*n* = 243, 72.3%) planned to travel to a single country, while 24% (*n* = 81) were visiting 2–3 countries. The remaining travellers (*n* = 11, 3.3%) planned to visit more than three countries on a single trip. The most popular single country of destination was India for 9.8% (*n* = 33) of older travellers. South Africa was the second most visited destination (*n* = 27, 8%), followed by Vietnam (*n* = 25, 7.45%), Brazil (*n* = 18, 5.3%), and Thailand (*n* = 16, 4.7%) (Fig. 1). South East Asia was the most popular geographic region with 39.2% (*n* = 132) of travellers. South America and Eastern Africa were the second most visited regions with 11.9% (*n* = 40) each, while Southern Africa and Western Africa received 8% (*n* = 27) and 3.9% (*n* = 13) of travellers, respectively (Fig. 2).

The majority of travellers (*n* = 267, 79.2%) had a documented pre-existing medical condition (Table 2). A total of 65 different illnesses were declared on their medical registration cards by the travellers in this study. The most commonly reported medical condition was hypertension at 7.7% (*n* = 26), followed by dyslipidaemia with 5.3% (*n* = 18), type 2 diabetes mellitus in 3.5% (*n* = 12), insect bite sensitivity affecting 3.2% (*n* = 11) and hypothyroidism 2.6% (*n* = 9) of travellers. The remaining illnesses comprised less than 1% of the sample including mental condition with 0.29% (*n* = 1). Travellers with no pre-existing medical condition represented only (*n* = 70, 20.8%) of the older travellers. There was no significant correlation between the healthier travellers and their destination (*p* = 0.09). More than half of the travellers (*n* = 211, 62.6%) reported that they are taking prescribed medications at the time of travel, while 36.8% (*n* = 124) were not taking any chronic medication. Antihypertensive agents (*n* = 32, 9.5%) and statins (*n* = 24, 7.1%) were the most frequently reported medications. Thyroxine (*n* = 15, 4.5%), inhalers (*n* = 9, 2.7%), and Vitamin D/Calcium (*n* = 5, 1.5%) were also frequently reported medications among the older travellers. Proton pump inhibitors (PPIs) at 0.3% (*n* = 1) were among the least reported medications (Table 3).

**Table 2:** Pre-existing medical conditions of older travellers

Pre-existing medical condition	Frequency (*N*)	Proportion (%)
Yes	267	79.2
No	70	20.7
Total	337	100
Type of Condition		
Others (including chronic cardiac, pulmonary and allergic condition)	191	56.6
Hypertension	26	7.7
Dyslipidaemia	18	5.3
Type 2 diabetes mellitus	12	3.5
Insect bite sensitivity	11	3.2
Hypothyroidism	9	2.6

**Table 3:** Chronic medication use by older travellers

Medication Type	Frequency (*N*)	Proportion (%)
Others (including anticoagulants, antiplatelets, analgesics and immunosuppressants)	124	36.7
Antihypertensive agents	32	9.4
Statins	24	7.1
Thyroxine	15	4.5
Inhalers	9	2.6
Vitamin D/Calcium	5	1.4

Most travellers (*n* = 245, 73.1%) had never received travel vaccines before their index clinic visit. Typhoid and hepatitis A were the most common vaccinations administered to travellers at the clinic in 33% (*n* = 112) and 25% (*n* = 84) of travellers, respectively. DPT (Diphtheria/Polio/Tetanus) in 19% (*n* = 65), yellow fever vaccine (*n* = 17, 5%), rabies vaccine (*n* = 15, 4.5%), quadrivalent meningococcal vaccine in 3.5% (*n* = 12), hepatitis B vaccine in 2.9% (*n* = 10) and oral cholera vaccine in 2.9% (n = 10) were the next most frequently prescribed vaccinations. Influenza (*n* = 4, 1.1%) and Japanese encephalitis (*n* = 2, 0.5%) were among the least frequently administered vaccines. Over a third (*n* = 122, 36.2%) of the older travellers were prescribed an antimalarial agent during their clinic visit, with atovaquone-proguanil being the most commonly prescribed antimalarial drug (*n* = 120, 35.6%) (Table 4).

**Table 4:** Prescribed vaccines and malaria chemoprophylaxis

Characteristics	Frequency (*N*)	Proportion (%)
Previous vaccine		
Yes	87	25.8
No	245	72.7
Vaccinations received at the clinic		
Typhoid	112	33.2
Hepatitis A	84	24.9
DPT (Diphtheria/Polio/Tetanus)	65	19.2
Yellow fever	17	5.0
Rabies	15	4.5
Meningococcal	12	3.5
Hepatitis B	10	2.9
Oral cholera	10	2.9
Influenza	4	1.1
Japanese encephalitis	2	0.5
Pneumococcal	0	0.0
Malaria chemoprophylaxis		
Atovaquone-Proguanil	120	35.6
Doxycycline	2	0.5

## Discussion

Our study is among the first of its kind to examine the demographics, travel characteristics and medical burden of older travellers in an attempt to help travel health professionals to provide more tailored pre-travel health advice. Most of the older travellers who visited the travel medicine clinic during the four-year period of data collection had at least one documented pre-existing medical illness on their travel registration card. We found that 79% of travellers had a documented medical condition, which is higher than the 38–74% reported in previous studies [19, 31, 32].

Alon and colleagues [31] found that the mean age of elderly travellers was 65.6 years with a nearly equal sex distribution. They also found that the most common medical condition among their elderly travellers was hypertension, followed by hyperlipidaemia and cardiovascular disorders. However, the authors concluded that older travellers were more compliant with medical advice than younger travellers, therefore, they were at lower risk for illness exacerbation during travel. Similarly, Stienlauf et al. [19] found that over half of older travellers who attended a pre-travel clinic in Israel between 2005 and 2007 had at least one pre-existing medical condition, with the most frequent co-morbidities including hypertension (26%), hyperlipaemia (23%) and diabetes (7%). The study concluded that the existence of a chronic medical condition does not have a significant impact on travel itinerary. However, they concluded that chronic use of medications may have an impact on travel duration. Hochberg and colleagues [32] found that 74% of older travellers attending five clinics in the greater Boston area in the USA between March 2008 and July 2010 had co-morbid medical conditions. The authors recommended that travellers with complex medical histories may warrant assessment by an experienced travel medicine physician.

Our study findings are comparable to earlier reports, in relation to demographics and the most frequently occurring comorbidities. The mean age of our study sample was 65 years old, with the most commonly reported pre-existing illnesses being hypertension, dyslipidaemia and endocrine disorders. This is particularly noteworthy as the current findings may reflect the true medical illness burden of international travellers despite the relatively small sample size. The prevalence of these pre-existing conditions is not surprising. Individuals with these conditions are often in a stable phase of the disease, thus may be more likely to choose to travel abroad [33, 34].

It was also of interest that the prevalence of diabetes mellitus among our elderly travellers was relatively low in this study. This may be due to the fact that diabetic travellers prefer to approach their own primary care doctor or endocrinologist, or perhaps they tend to avoid travelling overseas if they perceive their condition to pose too great a barrier to healthy travel. Several studies have shown that diabetic travellers are at higher risk of travel-associated illness, particularly infections and metabolic dysregulation [35–37], while other studies have reported more satisfactory outcomes for diabetics during travel abroad [38, 39].

The burden of psychiatric illness among our older travellers was also reported at a very low level (0.29%), compared to an earlier report (1.5%) [9]. This may be due to the low number of disclosures by travellers during their clinic registration. Any exacerbation of mental disorders during travel could present major challenges for the traveller, their companions as well as the local psychiatric health services at the host country which may be under resourced [40, 41].

The timing of the clinic visit before departure was 4 weeks in our older traveller cohort, which is adequate for travel physicians to provide appropriate intervention before travel. This interval allows the travel health specialists or general practitioners to counsel these high-risk travellers by optimising their pre-existing medical illness and titrating their medication dosage when necessary before their planned departure [42, 43].

In this study, India was the most popular country destination among elderly travellers, and Southeast Asia was the most popular single region despite its numerous travel health risks, including risks to personal safety, heat injury and vector-borne illnesses [44]. Our findings were similar to those of Swiss, Swedish and American studies [9, 44, 45], while Senegal and Sub-Saharan Africa were the top travel destinations in French and Spanish studies [46, 47]. One in ten of our travellers was travelling alone, which may put them at heightened risk of threats to their security and personal safety, as well as isolation from medical assistance in case of decompensation of their medical conditions [4].

More senior individuals taking chronic medication travel overseas; thus, travel medicine physicians may need to be more aware of issues surrounding geriatric medicine and polypharmacy [33]. Senior travellers frequently suffer from chronic diseases and take multiple medications; therefore, prescriptions by travel health specialists may lead to serious drug interactions or side effects. Few travellers in this study were taking anticoagulants; nonetheless, it is still a notable observation. Special consideration should be given to anticoagulant use during travel, particularly warfarin, which may give rise to potential drug-drug interactions in polypharmacy, or could have implications for malaria chemoprophylaxis, dietary factors and increased alcohol consumption, all of which could compromise bleeding control [48]. Travellers should also be encouraged to wear MedicAlert bracelets which state they are taking an anticoagulant [48, 49].

Travel to endemic vector-borne disease areas often necessitates the administration of vaccines and antimalarial chemoprophylaxis. Nearly the entire sample who visited the clinic were prescribed at least one vaccine and more that 30% received an antimalarial prescription. This may reflect the main reason for seeking consultation from the travel medicine clinic. This also implies that older travellers are more compliant in seeking pre-travel health care advice including vaccination and malaria chemoprophylaxis. This is in accordance with findings from other North American, European and Israeli studies [31, 50–53].

### Study limitations

The data from this study were limited by the fixed structure of the medical registration card used in our clinic, which had been designed many years previously and was not modified during the research period. At the clinic visit, the traveller was asked to complete the card without direct guidance, hence, there is a possibility of recall bias. However, this was often corrected during the subsequent consultation. As the data were self-reported, social desirability bias cannot be ruled out as a limitation of this study. Furthermore, some questions in the registration cards, for example, the purpose of travel, were insufficiently defined. In Particular the designation “other” should be sub-categorised. Recording more detailed travel itineraries (eg, urban vs rural area visit) would also help to facilitate a more comprehensive risk assessment. The travel medicine physician recorded further additional information regarding the travellers’ medical history on both their registration card and on a bespoke electronic medical record. Access to these electronic records was beyond the scope of the current research, however. Therefore, it is possible that the study population might have a higher burden of undocumented pre-existing medical conditions than what was apparent from the registration cards.

Our study findings may be extrapolated to older European travellers who seek pre-travel health advice. We believe that our study results provide insight into the characteristics, travel pattern, health conditions and pre-travel medical advice of a significant special group of travellers. Future prospective studies should investigate the travel risk-taking behaviour and care-seeking responses of older travellers during their travels abroad.

## Conclusions

This study increases our understanding of the demographics, travel characteristics and medical profile of a selected vulnerable group of travellers seeking advice at a specialist travel medicine clinic. As Ireland has a high level of outbound international travellers, the study findings are also of significance in an international context. A wide range of travel destinations, diseases and medication use was reported among this group of travellers, which could enable travel medicine physicians to provide more tailored advice and to select an appropriate pre-travel health intervention.

## Data Availability

The datasets during and/or analysed during the current study are available from the corresponding author.
